# Pneumonia-Induced Thyroid Crisis With Thyrotoxicosis Exacerbation: De Novo Graves' Disease Presentation on a Cornelia de Lange Syndrome (CdLS)

**DOI:** 10.7759/cureus.41119

**Published:** 2023-06-28

**Authors:** Jorge Pimentel Campillo, Daneybi Corona Castillo, Marco Nunez, Ana Finke Abbott, Jesús Pichardo

**Affiliations:** 1 Internal Medicine, Centros de Diagnóstico y Medicina Avanzada y de Conferencias Médicas y Telemedicina (CEDIMAT), Santo Domingo, DOM; 2 Endocrinology, Diabetes, and Metabolism, Centros de Diagnóstico y Medicina Avanzada y de Conferencias Médicas y Telemedicina (CEDIMAT), Santo Domingo, DOM; 3 Pediatrics, Icahn School of Medicine at Mount Sinai, New York, USA; 4 Internal Medicine, Norwalk Hospital, Norwalk, USA; 5 Surgery and Specialties, Centros de Diagnóstico y Medicina Avanzada y de Conferencias Médicas y Telemedicina (CEDIMAT), Santo Domingo, DOM

**Keywords:** cornelia de lange, pneumonia, internal medicine, pancytopenia, thyrotoxicosis, infectious diseases, metabolism, endocrinology, genetics, congenital disease

## Abstract

Cornelia de Lange syndrome (CdLS) is a rare genetic disorder that affects multiple organ systems and is characterized by distinctive facial features, growth retardation, and developmental disabilities. The syndrome is caused by mutations in genes (*NIPBL*, *RAD21*, *SMC3*, *HDAC8*, and *SMC1A*) involved in the cohesin complex, which plays a critical role in chromosome segregation and gene expression regulation. Clinical findings typically include dysmorphic facial features (arched eyebrows, synophrys, long eyelashes, ptosis, long philtrum, thin upper lip, and posteriorly rotated ears), growth and mental retardation, upper limb defects (clinodactyly and limb deficiencies), gastrointestinal complications (gastroesophageal reflux, pyloric stenosis, diaphragmatic hernia, malrotation, and volvulus), and heart defects (ventricular and atrial septal defects). In addition, chronic respiratory tract infections including sinusitis and pneumonia have been frequently described in this population. The early recognition and diagnosis of CdLS through genetic testing are crucial to ensure appropriate medical management and early intervention therapies to improve the outcomes of affected individuals.

The thyroid gland is not affected by this congenital disease, but complications can arrive in this organ by other disease-related organ malfunctions. Pneumonia-induced thyroiditis is a potentially life-threatening condition that can occur in patients with underlying thyroid disease who also develop pneumonia. The symptoms are due to the hypermetabolic state induced by excess thyroid hormones and include weight loss, heat intolerance, and palpitations. There are many different causes of thyrotoxicosis. It is important to determine the cause since treatment is based on the underlying etiology. The diagnosis of pneumonia-induced thyrotoxicosis can be challenging as symptoms may mimic other conditions and laboratory testing may not always provide a clear answer. The diagnosis is confirmed with low thyroid-stimulating hormone (TSH) and elevated free thyroxine (T4) and triiodothyronine (T3), erythrocyte sedimentation rate (ESR), and C-reactive protein.

The management of the condition involves prompt recognition, supportive care, and the use of medications to lower thyroid hormone levels, such as beta-blockers, antithyroid drugs, steroids, and iodine. In severe cases, plasmapheresis or thyroidectomy may be necessary.

## Introduction

Cornelia de Lange syndrome (CdLS) is a very rare syndrome characterized by multiple congenital anomalies affecting various organs and severe mental retardation. Its incidence has been reported to be 1:10,000-20,000 among the general population, with no racial predilection [1]. The syndrome can arise due to multifactorial mutations in the genes *NIPBL* on chromosome 5, *RAD21* on chromosome 8, *SMC3* on chromosome 10, and *HDAC8* and *SMC1A* in chromosome X, which are involved in the cohesin complex, which plays a critical role in chromosome segregation and gene expression regulation [1]. It shows distinctive facial features, including arched eyebrows, synophrys (the fusion of the eyebrows in the midline), long eyelashes, a small nose, and a thin upper lip [2]. Individuals with CdLS also typically have growth retardation, microcephaly, and developmental disabilities, including intellectual disability and delays in speech and motor skills. Other common features of CdLS include gastroesophageal reflux disease (GERD), feeding difficulties, seizures, and hearing loss [3].

CdLS has a variable clinical presentation and can affect individuals differently, even within the same family. There are three types of CdLS: classic, mild, and severe. The early recognition and diagnosis of CdLS are crucial to ensure appropriate medical management and early intervention therapies to improve the outcomes of affected individuals [4]. The treatment of CdLS is focused on managing medical complications, providing supportive care, and maximizing the individual's potential for development and independence. A multidisciplinary approach, involving healthcare professionals from various specialties, is typically required to provide comprehensive care for individuals with CdLS. Furthermore, individuals in this group often experience persistent respiratory tract infections such as sinusitis and pneumonia. Notably, a substantial study investigating mortality causes within this syndrome revealed that approximately 13% of deaths were attributed to respiratory failure caused by infections and sepsis. The frequent occurrence of infections in CdLS patients is commonly attributed to concurrent anatomical abnormalities, including cleft palate, severe gastroesophageal reflux, and narrow ear canals [5]. By itself, CdLS does not present with endocrinologic abnormalities, but these infections can make other conditions flare up. One of those is Graves' disease, which can cause thyrotoxicosis in a known patient who develops pneumonia.

Pneumonia-induced thyroiditis refers to the inflammation of the thyroid gland that occurs as a result of a severe respiratory infection such as pneumonia. This condition occurs when the infection spreads to the thyroid gland, leading to its inflammation and dysfunction. Pneumonia-induced thyroiditis can manifest with symptoms such as fever, neck pain, swelling, and difficulty swallowing. In some cases, it can also result in the release of thyroid hormones into the bloodstream, leading to hyperthyroidism. Thyroid storm, also referred to as thyroid or thyrotoxic crisis, is a rare medical condition that represents an extremely severe physiological state within the range of thyrotoxicosis. Although uncommon, it is important to note that the mortality rates associated with this condition are alarmingly high, potentially reaching to levels as high as 10%-20% [5]. Thyroid storm is usually associated with Graves' hyperthyroidism but can also occur in any form of thyrotoxicosis. It is characterized by organ failure symptoms, with fever being a common symptom. The treatment primarily focuses on providing support through cooling and fluid administration. Additionally, steps are taken to decrease the production and release of thyroid hormones, as well as counteract the excessive effects of these hormones on the body. It is important to also identify and treat any factors that may have triggered the thyroid storm.

## Case presentation

**Figure 1:**
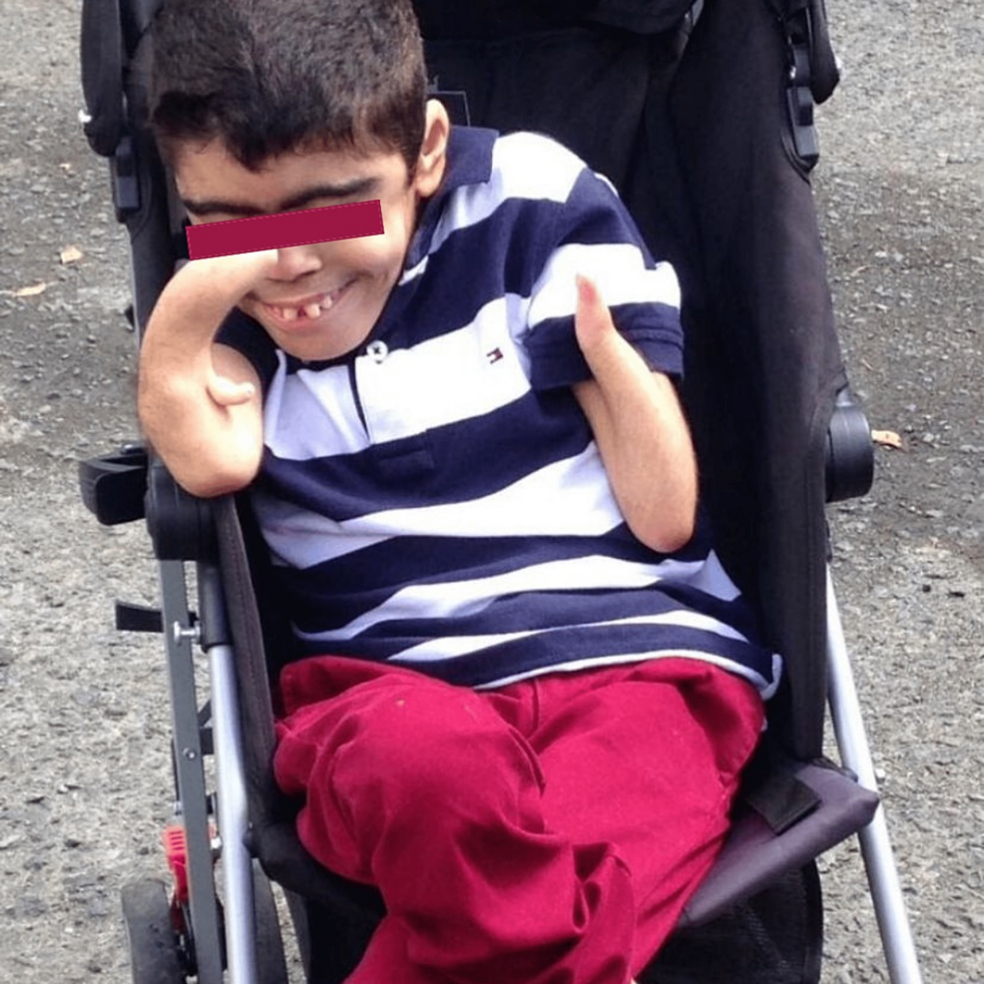
Physical features of the 20-year-old patient with Cornelia de Lange syndrome.

**Figure 2:**
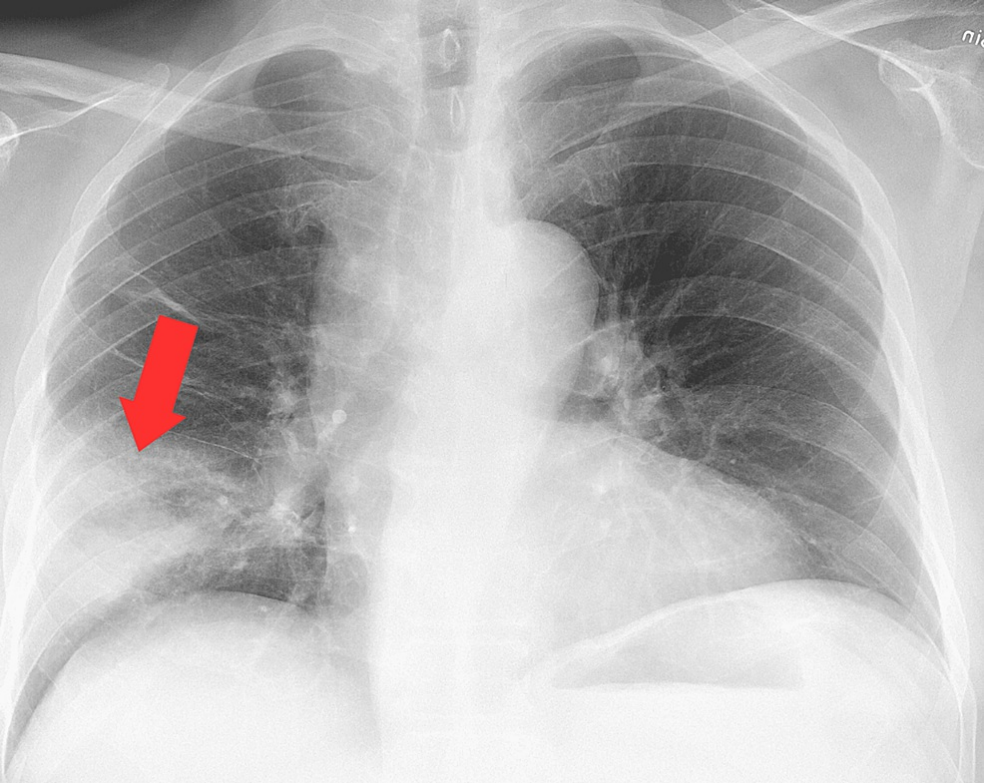
Chest X-ray. Red arrow showing middle lobe pneumonia.

In this report, we present a 20-year-old male diagnosed with CdLS (Figure 1) at birth with recurrent aspiration-induced pneumonia and no known history of thyroid disease, who was taken by his mother to the emergency room due to high fever, dyspnea, and drowsiness for the past two days. He was diagnosed with pneumonia based on clinical and radiological findings and was started on empiric ampicillin/sulbactam antibiotics (Figure 2).

However, within 24 hours of admission, the patient developed tachycardia, hypertension, and confusion. Physical examination revealed a temperature of 39°C, pulse rate of 140 beats per minute, blood pressure of 180/100 mmHg, and a Glasgow Coma Scale score of 12. Thyroid examination was unremarkable since pain response could not be evaluated. Laboratory investigations revealed elevated levels of free thyroxine (T4) and triiodothyronine (T3) and low thyroid-stimulating hormone (TSH) (Table 1).

**Table 1:** Laboratory values of the patient showing the difference from admission date to a week after treatment. T4, thyroxine; T3, triiodothyronine

Test (serum)	Day of admission	Week after treatment	
Free T3 (normal range: 0.26-0.48 ng/dL)	8.32 ng/dL (high)	2.21 ng/dL (high but lower)	
Free T4 (normal range: 0.8-2.0 ng/dL)	9.65 ng/dL (high)	2.87 ng/dL (high but lower)	
Thyroid-stimulating hormone (TSH) (normal range: 0.5-5 mlU/L)	0.05 mlU/L (low)	1.87 mlU/L (normal)	
Red blood cell (RBC) (normal range: 4.7-6.1 mm cells/mcL)	5.1 cells/mcL (normal)	5.6 cells/mcL (normal)	
Hemoglobin (Hb) (normal range: 13.8-17.2 g/dL)	14.2 g/dL (normal)	14.8 g/dL (normal)	
White blood cell (RBC) (normal range: 4.5-11.0 × 10^9^/L)	19 × 10^9^/L (high)		10.5 × 10^9^/L (normal)
Platelets (normal range: 150-400 × 10^9^/L)	325 × 10^9^/L (normal)		315 × 10^9^/L (normal)

Since the patient has a positive family history of Graves' disease from his mother, further laboratory testing was indicated including thyroid-stimulating receptor antibody (anti-TSH), which came back positive (Table 2).

**Table 2:** Result of thyroid receptor antibody (TRAb).

Test (serum)	Result
Thyroid receptor antibody (TRAb) (normal value: 0-0.9 IU/L)	12.4 IU/L (high)

Based on the clinical presentation and laboratory findings, a diagnosis of pneumonia-induced thyroiditis was made. The patient was started on prednisone, atenolol, and propylthiouracil (PTU) in addition to the previously mentioned antibiotics. He was also given supportive care, including intravenous fluids and electrolyte replacement. Within 24 hours of starting treatment, the patient's vital signs stabilized, and his level of consciousness improved.

After a week of hospitalization, the patient was discharged with a prescription for the same in-patient medications. The patient's mother was advised to follow up with an endocrinologist and continue treatment for Graves' disease.

## Discussion

Cornelia de Lange syndrome (CdLS) is a rare genetic disorder that affects multiple organs and systems, including the face, limbs, heart, and gastrointestinal tract. It is caused by mutations in one of several genes involved in the regulation of chromatin and gene expression during embryonic development [2]. The disorder is characterized by cardinal facial features (thick eyebrows, synophrys, concave nasal ridge, upturned nasal tip, short nose, thin upper lip vermilion, and downturned corners of the mouth), small stature, intellectual disability, and behavioral problems [4]. Our case report describes a patient with CdLS who presented with recurrent respiratory infections and gastrointestinal symptoms. The patient had the characteristic facial features and small stature seen in CdLS, as well as severe developmental delay and behavioral problems. It was previously diagnosed at birth by fluorescence in situ hybridization (FISH) demonstrating a microdeletion of chromosome 5 affecting the *NIPBL *gene. The *NIPBL *gene provides instructions for making the protein called delangin, which plays an important role in human development. Delangin helps control the activity of chromosomes during cell division [6]. The management of CdLS is primarily supportive and multidisciplinary, involving a team of specialists such as pediatricians, geneticists, gastroenterologists, and developmental pediatricians [1]. Early intervention programs that focus on communication, behavioral, and cognitive therapies can improve outcomes in patients with CdLS. Additionally, prompt treatment of respiratory and gastrointestinal complications is essential to prevent morbidity and mortality.

Several recent studies have shed light on the genetic and molecular mechanisms underlying CdLS. These studies have identified several genes involved in the regulation of chromatin and gene expression that are mutated in CdLS. These findings have led to the development of targeted therapies that aim to correct the molecular defects associated with CdLS. For example, a recent study demonstrated that treatment with a small molecule that inhibits a protein called BRD4 can partially rescue the developmental defects associated with CdLS in a mouse model [7].

Respiratory infections are a common cause of morbidity and mortality in patients with CdLS. They can be due to multiple factors, including immune dysfunction, upper airway abnormalities, and aspiration. Gastrointestinal symptoms such as feeding difficulties, gastroesophageal reflux disease, and constipation are also common in CdLS and can lead to malnutrition and growth failure [8].

The endocrine system is not commonly affected by CdLS, but the exacerbation of undiagnosed conditions can arise as complications of this genetic disorder. Recurrent aspiration pneumonia due to acid reflux and oropharyngeal muscle dysfunction is the primary cause of pneumonia in CdLS patients; therefore, conditions that may get worse by the body's physiological response to infections will arise. This patient has a family history of Graves' disease from his mother but never presented thyroid problems until that moment when we concluded that recurrent infections led to the exacerbation of undiagnosed Graves' disease. After appropriate diagnostic tests as described before, Graves' disease diagnosis was established.

Pneumonia-induced thyroiditis is a rare but potentially life-threatening condition when it evolves into thyrotoxicosis, which can occur in patients with underlying thyroid disease who also develop pneumonia. In the United States, the incidence of thyrotoxicosis ranges from 0.57 to 0.76 cases per 100,000 persons per year [9]. The condition is characterized by a rapid and severe increase in thyroid hormone levels, resulting in a range of symptoms including fever, tachycardia, hypertension, delirium, and even coma. There are several proposed mechanisms for how pneumonia can trigger thyrotoxicosis, including increased cytokine production, the stress-induced activation of the hypothalamic-pituitary-thyroid axis, and the exacerbation of underlying thyroid disease [10-12].

The management of the condition involves prompt recognition, supportive care, and the use of medications to lower thyroid hormone levels, such as beta-blockers, steroids, antithyroid drugs, and iodine. In severe cases, plasmapheresis or thyroidectomy may be necessary [10].

Our case report highlights the importance of the early recognition and management of respiratory and endocrinologic complications in patients with CdLS. The management of this disorder is primarily supportive and multidisciplinary, involving a team of specialists. Advances in the understanding of the genetic and molecular mechanisms underlying CdLS have opened up new avenues for the development of targeted therapies, which hold promise for improving outcomes in patients with this challenging disorder.

## Conclusions

Patients with Cornelia de Lange syndrome (CdLS) are at an increased risk of respiratory infections due to upper airway abnormalities, immune dysfunction, and aspiration. The respiratory manifestations of CdLS can be severe and may lead to morbidity and mortality. Our case report highlights the importance of the early recognition and management of respiratory infections in patients with CdLS. The timely administration of appropriate antibiotics and supportive care can prevent complications and improve outcomes. In addition, a multidisciplinary approach involving a team of specialists is essential to address the multiple organ and system involvement seen in CdLS. Advances in the understanding of the genetic and molecular mechanisms underlying CdLS hold promise for the development of targeted therapies that can improve outcomes in patients with this challenging disorder. Further research is needed to elucidate the mechanisms underlying respiratory infections in CdLS and to develop more effective strategies for their prevention and treatment.
